# Two new species of *Molophilus* Curtis from the Mediterranean and Transcaucasia (Diptera, Limoniidae)

**DOI:** 10.3897/zookeys.871.34559

**Published:** 2019-08-12

**Authors:** Jaroslav Starý, Jozef Oboňa

**Affiliations:** 1 Neklanova 7, CZ-779 00 Olomouc-Nedvězí & Silesian Museum, Nádražní okruh 31, CZ-746 01 Opava, Czech Republic Olomouc-Nedvězí & Silesian Museum Opava Czech Republic; 2 Department of Ecology, Faculty of Humanities and Natural Sciences, University of Prešov, 17. novembra 1, SK-081 16 Prešov, Slovakia University of Prešov Prešov Slovakia

**Keywords:** Limoniid Crane Flies, West Palaearctic Molophilus (Molophilus), male terminalia, description

## Abstract

Two new species of the genus *Molophilus* Curtis, 1833 are described, Molophilus (Molophilus) rohaceki**sp. nov.** (Italy: Calabria) and M. (M.) soldani**sp. nov.** (Azerbaijan), and their male terminalia are illustrated.

## Introduction

The genus *Molophilus* Curtis, 1833 is a cosmopolitan taxon (101 West Palaearctic species, 1018 species worldwide in 11 subgenera, cf. Oosterbroek 2019) and new species are still named even from well-investigated territories. Recently, a paper was published with descriptions, redescriptions, and other nomenclatural changes, dealing principally with the west Palaearctic Molophilus (Molophilus) species (Starý 2011). As a minor addition to this species-richness, another two west Palaearctic species are described here, M. (M.) rohaceki sp. nov. (Italy: Calabria) and M. (M.) soldani sp. nov. (Azerbaijan). The two species are distantly related to each other, both belonging to the same morphological group distinguished by a comparatively simple shape of the dorsal portion of the gonocoxite, having no dorsal and/or lateral lobes. Within this group numerous west Palaearctic species are known, such as M. (M.) undulatus Tonnoir, 1920, M. (M.) scutellatus Goetghebuer, 1929, M. (M.) brevihamatus Bangerter, 1947, and many others, sometimes variously modified in the lateral outline of the dorsal portion of the gonocoxite. However, the new species differ from their consubgeners and from each other considerably in various distinctive features. They are somewhat exceptional in having their aedeagal plates smooth, without any microsetae.

## Materials and methods

**Figures 1–4.**
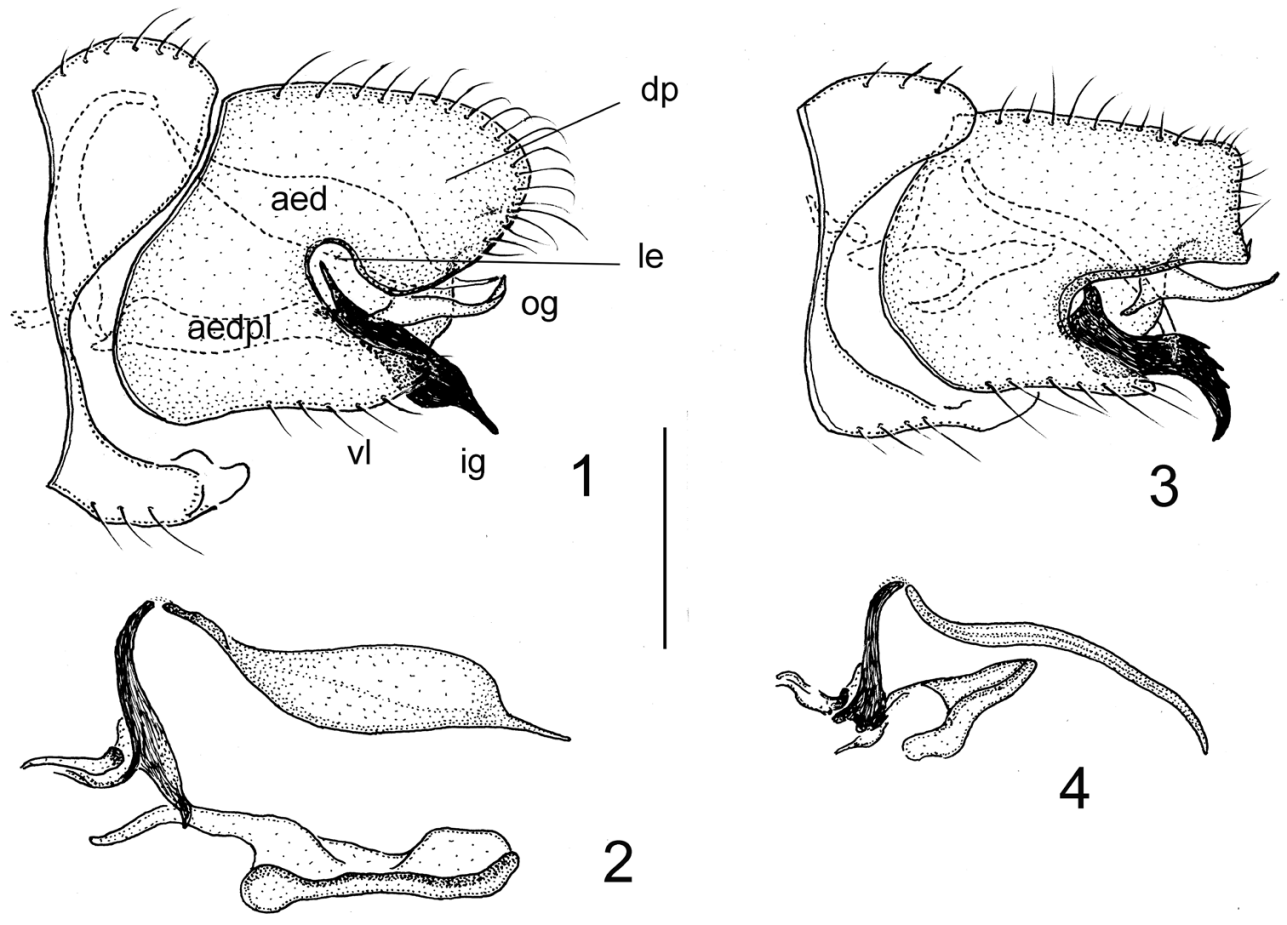
Male terminalia. **1–2**M. (M.) rohaceki sp. nov. (holotype) **1** general view, lateral **2** aedeagal complex, lateral **3–4**M. (M.) soldani sp. nov. (holotype) **3** general view, lateral **4** aedeagal complex, lateral. Scale bar: 0.25 mm. Abbreviations: aed – aedeagus; aedpl – aedeagal plate; dp – dorsal portion of gonocoxite; ig – inner gonostylus; le – lateral excision; og – outer gonostylus; vl – ventral lobe of gonocoxite.

The morphological terminology adopted here follows essentially that of McAlpine (1981). Some special terms of structures of the *Molophilus* male terminalia are referred to in Fig. 1.

All type specimens are preserved dry, glued on cardboard points. Since the specimens of M. (M.) soldani sp. nov. were dried from ethanol, the colour features as indicated in the description may differ somewhat from a normally dried state. The male terminalia were preserved in glycerine in a small plastic tube pinned below the associated specimen.

The following museum and collection acronyms are used in the text:

**JSO** Collection of J. Starý, Olomouc, Czech Republic;

**SMOC**Slezské zemské museum (Silesian Museum), Opava, Czech Republic.

### Molophilus (Molophilus) rohaceki sp. nov.

Taxon classificationAnimaliaDipteraLimoniidae

97cef190-32f1-5528-8111-24792cb2b3bc

http://zoobank.org/611A0D7B-2671-4E51-B2F9-5CA0A328E575

Figs 1 , 2

#### Diagnosis.

Medium-sized (wing length 4–5 mm) species within *Molophilus*. Body deep dark brown, almost black, mostly shiny, locally suffused with slight greyish pruinosity, restrictedly patterned with yellow. Male terminalia with outer (dorsal) gonostylus pale, inner (ventral) gonostylus darkly pigmented, latter longer than former. Aedeagus exceedingly swollen for most of its length, except for slender terminal portion. Aedeagal plate long, rectangular in ventral aspect, without microsetae, level with base of terminal portion of aedeagus.

#### Description.

***Male.*** Head. Antenna slightly lengthened, compared to most other west Palaearctic species, extending beyond wing base, dark brown throughout. Flagellomeres long-ovoid, with longest verticils subequal to length of their respective segments.

Thorax in general deep dark brown to black, mostly shiny. Prescutum and scutum mostly black, shiny, with slight greyish pruinosity and slightly paler laterally, yellowed lateral to prescutal pit and around wing base, pale yellow on paratergite anteriorly. Scutellum and postscutellum shiny black, paler laterally. Pleuron shiny black throughout. Wing length 5.0 mm. Wing membrane slightly infuscate; venation as for genus. Halter yellowish brown. Legs yellowish brown, with tips of femora and tarsi darkened.

Abdomen deep dark brown to black. Male terminalia (Figs 1, 2) black. Dorsal portion of gonocoxite rather short and broad, broadly rounded in lateral aspect. Lateral excision deep and moderately wide, compared to similar species. Ventral lobe of gonocoxite broad and rather short, its rounded tip not reaching apex of dorsal portion. Both gonostyli slightly arched medially. Outer gonostylus pale, generally slender, slightly upturned, narrowly obtuse at apex, extending to ca. three fourths length of inner gonostylus. Inner gonostylus darkly pigmented, powerful, reaching beyond ventral lobe by ca. one third its length or more, gradually broadened in lateral aspect, then abruptly tapered into slender, slightly upturned and obliquely truncate apical part. Aedeagus moderate in length, exceedingly swollen for most of its length, abruptly tapered into slender terminal portion, subacute at tip. Aedeagal plate smooth, without microsetae, rectangular in ventral aspect, when preserved under natural configuration of other structures of aedeagal complex, level with base of terminal portion of aedeagus (Fig. 2).

***Female.*** Unknown.

#### Material examined.

***Holotype*** ♂: Greece, SW Peloponnese, Taygetos Mts, Alagonia 2.4 km NW (1335 m), 37°06'55"N, 22°16'07"E, brook, springs, 9.10.2017 (J. Roháček leg.) (SMOC).

#### Etymology.

The new species is named in honour of its collector, Dr. Jindřich Roháček (Silesian Museum, Opava, Czech Republic), a world-famous specialist of Anthomyzidae and Sphaeroceridae (Diptera). A noun in genitive singular.

#### Discussion.

The new species differs from its similar congeners by details in the structure of the male terminalia, especially its swollen aedeagus and very long aedeagal plate. Molophilus (M.) brevihamatus Bangerter, 1947 has a similarly swollen aedeagus, but is distinctive by various other features, including body colouration (brown in M. (M.) brevihamatus, shiny black in the new species) and shape of gonostyli and aedeagal plate.

### Molophilus (Molophilus) soldani sp. nov.

Taxon classificationAnimaliaDipteraLimoniidae

8471608a-b925-5abf-80a1-cb3572272065

http://zoobank.org/4CF8ABCD-5B04-4774-8CDD-5426298C15FF

Figs 3 , 4

#### Diagnosis.

Medium-sized species within *Molophilus*. Body dark brown, suffused with dense greyish pruinosity, restrictedly patterned with yellow. Male terminalia with outer (dorsal) gonostylus pale, inner (ventral) gonostylus darkly pigmented, latter longer than former, with three distinct teeth on dorsal surface. Aedeagus slender, sinuous, with its tip down-curved. Aedeagal plate short, triangular in ventral aspect, without microsetae, with its tip pointing to ca. one third length of aedeagus.

#### Description.

***Male.*** Head. Antenna of both holotype and male paratype broken off, but probably much same as that of female paratype, of moderate length, extending to approximately wing base, dark brown throughout. Flagellomeres ovoid, with longest verticils subequal to length of their respective segments.

Thorax in general dark greyish brown. Prescutum and scutum mostly dark brown, suffused with dense greyish pruinosity, slightly paler laterally, yellowed lateral to prescutal pit and around wing base, pale yellow on paratergite anteriorly. Scutellum yellow, dark greyish brown anteriorly, postscutellum dark greyish brown, narrowly yellowed laterally. Pleuron mostly dark greyish brown, restrictedly paler, especially on anepimeron. Wing length 4.2–4.9 mm. Wing membrane slightly infuscate; venation as for genus. Halter pale yellow. Legs yellowish brown, with tips of femora indistinctly darkened and tarsi dark brown.

Abdomen dark greyish brown. Male terminalia (Figs 3–4) dark brown. Dorsal portion of gonocoxite moderately long, quadrangular in lateral aspect, slightly narrowed distally, almost straight at posterior margin, with tiny membranous process at ventral corner. Lateral excision moderately deep and rather wide. Ventral lobe of gonocoxite moderately broad and rather short, its narrowly rounded apex extended shortly beyond base of outer gonostylus. Both gonostyli slightly arched medially. Outer gonostylus pale, gradually tapered distally into slender, obtuse tip, extending to ca. three fourths length of inner gonostylus. The latter darkly pigmented, powerful, sharply sinuous, exceeding ventral lobe by ca. two thirds its length, gradually tapered at ca. mid-length into slender distal part, with three distinct teeth on dorsal surface. Aedeagus moderate in length, generally slender, slightly sinuous, with its tip down-curved. Aedeagal plate smooth, without any microsetae, generally triangular in ventral aspect, when preserved under natural configuration of other structures of aedeagal complex, pointing with its rounded apex to ca. one third length of aedeagus (Fig. 4).

***Female.*** Female resembling male in general appearance. Female terminalia with cercus very slender, gently upturned, subacute at tip, approximately twice length of tergite 10. Hypogynal valve straight, reaching to ca. three fourths length of cercus.

#### Material examined.

***Holotype*** ♂: Azerbaijan, Qabala district, S of Durca, nr. tributary of Qaraschay R. (1236 m), 41°02'11.2"N, 47°53'13.6"E (light trap), 30.5.2017 (Ľ. Hrivniak leg.) (SMOC). ***Paratypes***: 1 ♂, 1 ♀, same data as for holotype (JSO).

#### Etymology.

The new species is named in honour of the late Prof. Dr. Tomáš Soldán, an outstanding Ephemeroptera specialist. A noun in genitive singular.

#### Discussion.

The new species is distinctive by the combination of the quadrangular outline of its dorsal portion of gonocoxite and an unusually shaped inner gonostylus with three distinct teeth on the dorsal surface. The type specimens were dried from ethanol; hence, their body colouration might be darker in a normal dried state.

## Supplementary Material

XML Treatment for Molophilus (Molophilus) rohaceki

XML Treatment for Molophilus (Molophilus) soldani
